# Comparison of radiofrequency ablation and partial nephrectomy for tumor in a solitary kidney

**DOI:** 10.1186/s12894-017-0269-4

**Published:** 2017-09-06

**Authors:** Wu Xiaobing, Gong Wentao, Liu Guangxiang, Zhang Fan, Gan Weidong, Guo Hongqian, Zhang Gutian

**Affiliations:** Nanjing University Medical School Affiliated Nanjing Drum Tower Hospital, Nanjing, 210008 China

**Keywords:** Radiofrequency ablation, Partial nephrectomy, Solitary kidney, Oncologic and functional outcomes

## Abstract

**Background:**

To estimate oncologic and functional outcomes for radiofrequency ablation (RFA) versus partial nephrectomy (PN) for tumors in a solitary kidney.

**Methods:**

Nineteen patients with sporadic renal cell carcinoma in a solitary kidney were treated with RFA, and 21 patients were treated with PN between November 2008 and September 2015. Basic demographic information including age, gender, operative and pathological data, complications, renal function, oncological outcomes, was obtained for each patient. Statistical analysis was done to test for the correlation of clinical and pathological features, renal function outcomes, as well as oncological outcomes of RFA and PN. All statistical tests were 2-sided, and *p*-value < 0.05 was considered significant. Statistical analyses were performed using SPSS 19.0.

**Results:**

No significant differences were indicated between the RFA and PN with respect to mean patient age, tumor size, as well as intraoperative or postoperative complications. The mean length of hospitalization (*P* = 0.019) and mean operative time (*P* = 0.036) was significantly shorter in RFA, with the median estimated blood loss being greater in PN (*P* = 0.001). The mean serum creatinine level 24 h following operation were significantly higher than preoperative creatinine in PN (*P* = 0.009), but did not reach statistical significance in RFA. Local recurrence were detected in only 1 patient (5%) in PN and 3 patients (18.75%) in RFA (*P* = 0.4). One patient developed pulmonary metastasis and one exhibited tumor persistence in RFA, none were present in PN.

**Conclusions:**

Radiofrequency Ablation and Partial Nephrectomy for Tumors in a Solitary Kidney were all safe and effective, with each method having distinct advantages. It is the decision of the patient and urologist to pick the best approach.

## Background

The incidence of renal cell carcinoma (RCC) continues to increase because of the widespread use of modern imaging examination, with an increasing number of patients with no urological symptoms being examined. Radical nephrectomy (RN) used to be regarded as the gold standard for the treatment of RCC for many years; recently, nephron-sparing surgery (NSS) was recommended particularly for tumors in a solitary kidney by a number of researchers and specialists. Tumors in a solitary kidney represent a challenging population where tumor control with maximal nephron preservation is essential. RN for tumors in a solitary kidney could lead to permanent hematodialysis; open partial nephrectomy (OPN) was regarded as the gold standard therapy for non-metastatic tumors in a solitary kidney, and laparoscopic partial nephrectomy (LPN) for tumor in a solitary kidney was feasible [1]. Radiofrequency ablation (RFA) was also considered as an approach to RCC [2]. Both PN (partial nephrectomy) and RFA (radiofrequency ablation) could be used for tumors in a solitary kidney. This study aimed to evaluate oncologic and functional outcomes for RFA versus PN for tumors in a solitary kidney, thus helping patients decide on the suitable surgery.

## Methods

### Patients

The Nanjing Drum Tower Hospital Urologic Oncology Database, which was approved by the Institutional Review Board, was retrospectively reviewed. Nineteen patients with RCC in a solitary kidney were treated with RFA, and 21 patients were treated with PN between November 2008 and September 2015. Patients with confirmed single or multiple tumors in an anatomical (congenital or acquired) or functional solitary kidney shown in the CT or MRI of the urinary tract were selected. Patients presenting with bilateral metastasis or have a history of hereditary RCC or a family history of RCC were excluded from analysis. One patient in the PN group and 3 patients in the RFA group who had metastasis preoperatively and received palliative therapy (via oral medication) were subsequently excluded from our analysis.

### Surgical methods

Approaches to RFA include percutaneous and laparoscopic procedures; these surgical techniques for RFA have been described in previous studies [3, 4]. In the present study, the Cool-tip RFA system was used; all RFAs were conducted by the same experienced physician, and all cases followed the protocol provided by the system manufacturer. During RFA, ultrasound localization was conducted to locate endophytic tumors and ensure that the tumors were completely ablated; ablation cycles depended on tumor size. Renal biopsies were not obtained for every patient who received RFA.

Approaches to PN include open or laparoscopic surgery (LPN and RPN); the surgical techniques have been previously described [5–7]. After clamping of the renal artery during PN, the tumor was excised sharply outside the zone of a 0.5 cm peritumoral margin. The wound was sutured, and the collecting systems were closed when necessary.

### Recorded variables and follow-up

Follow-up data was collected up to March 2016. The recorded variables included the following: patient age, gender, tumor size, R.E.N.A.L. nephrometry score, operative time, estimated blood loss (EBL), intraoperative and postoperative complications, length of hospitalization, pathologic outcomes, and serum creatinine. Post-operative follow-up included the follow-up period, recurrence, and metastasis. Complications were classified as intraoperative and postoperative. Intraoperative complications included significant injury to an adjacent organ, major vessel, ureter or pleura, and conversion to open surgery or transfusion (> 2 units). Post-operative complications included serious infection (requiring antibiotics), urine leakage, hemorrhage (requiring second surgery to stop bleeding). The R.E.N.A.L. nephrometry scoring system comprises the tumor size, tumor depth, proximity to the collecting system, tumor positioning in the anterior/posterior plane, and tumor location with regard to polarity; this system was used to measure the comorbidity in patients with renal tumors [8].

For RFA and PN, these data were obtained at 3 months, 12 months, and yearly thereafter; patients were contacted via outpatient review and telephone.

### Statistical methods

Descriptive statistics are presented as the mean ± SD, median, and range or percentage. Demographic and clinical characteristics were analyzed using Student’s t-tests for continuous variables and the chi-square test for categorical variables. The survival curve was generated using the Kaplan–Meier method, and differences between 2 groups were assessed using the log-rank test. All statistical tests were 2-sided, and *p*-value < 0.05 was considered significant. Statistical analyses were performed using SPSS 19.0.

## Results

A total of 20 patients in the PN group and 16 patients in the RFA group satisfied the selection criteria. Demographic and clinical characteristics are presented in Table 1. No significant differences were indicated between the RFA and PN with respect to mean patient age, tumor size, as well as intraoperative or postoperative complications. The tumor complexity of the RFA group was similar to that of the PN group (*p* = 0.56). PN was associated with a greater median EBL (377 vs. 65.6 mL; *p* = 0.001). The mean length of hospitalization (12 vs. 17 d; *p* = 0.019) and mean operative time (145 vs. 199.8 min; *p* = 0.036) were significantly shorter in the RFA group. Mean renal artery blocking time was 25.2 min in the PN group, whereas no hilar clamping was necessary in the RFA group. Intraoperative (23.8% vs. 5.6%; *p* = 0.13) and postoperative complications (19.0% vs. 11.1%; *p* = 0.41) tended to be higher in the PN group; similarly, total complications were higher (42.8% vs. 16.7%; *p* = 0.096), but no significant difference was determined.

**Table 1:** Demographic and clinical characteristics

	RFA	PN	*P* value
Patients	16	20	
Procedures	18	21	–
Male/female	11/5	15/5	0.48
Mean age(years)(range)	59.6(36–79)	62.1(41–83)	0.55
Left/right	10/8	13/8	0.47
Tumor size (cm) (range)	3.4(1.5–6.0)	3.6(1.5–6.0)	0.5
Median EBL (mL) (range)	65.6(20–200)	377(20–1200)	0.001
Surgical approach, n (%)			
Percutaneous	7	–	–
Laparoscopic	7	11	–
Open	2	9	–
Mean operative time (min) (range)	145.0(50–260)	199.8(60–440)	0.036
Mean length of stay (days) (range)	12(6–28)	17(9–42)	0.019
renal artery blocking time (min)	–	25.2	–
complications (%)	3(16.7)	9(42.8)	0.096
Intra-operative complications	1(5.6)	5(23.8)	0.13
Postoperative complications	2(11.1)	4(19.0)	0.41

Renal function outcomes are presented in Table 2. The mean serum creatinine level (120.4 μmol/L) was significantly higher 24 h post-operatively than preoperatively (91.4 μmol/L) in the PN group (*p* = 0.01). In the RFA group, mean serum creatinine level (108.5 μmol/L) trended to higher 24 h post-operatively than preoperatively (94.9 μmol/L); however, no significant difference was indicated (*p* = 0.06). Three months post-operatively, the mean serum creatinine level in each group showed no significant increase (*p* = 0.26; *p* = 0.45). No dialysis was needed for either group during the follow-up period.

**Table 2:** Renal function data

Mean serum creatinine (μmol/L)(range)	Pre-operation	24 h following operation	3 mon after operation	P1	P2
PN group	91.4(53–174)	120.4(75–209)	102.3(50–200)	0.01	0.26
RFA group	94.9(66–113)	108.5(80–154)	98.7(70.6–120)	0.06	0.45

P1: 24 h following operation vs. pre-operation in each group; P2: 3 months after operation vs. pre-operation in each group

Oncologic outcomes are listed in Table 3. In the PN group, no incidence of positive surgical margins was reported. Mean follow-up was 26.1 months in the PN group and 33.7 months in the RFA group (*p* = 0.25). In the PN group, 18 cases were identified as RCC: 1 was papillary, and 2 were chromophobe. In the RFA group, 14 cases were identified as RCC, and four were not specified. Local recurrence was detected in only one patient (5%) in the PN group and three patients (18.75%) in the RFA group (*p* = 0.1). Overall, one of 16 patients (6.25%) in the RFA group had incomplete ablation; the patient was successfully re-ablated 8 months later and remained cancer-free. In addition, one patient developed multiple metastasis in the RFA group, where none was reported in the PN group. Table 4 presents detailed information on recurrence; no deaths were reported.

**Table 3:** Oncological outcome

	PN	RFA	*P* value
No. patients	20	16	
Procedures	21	18	–
Tumor histology, n (%)			
Clear cell	18	14	
Papillary	1	0	
Chromophobe	2	0	–
Not specified	0	4	
Positive surgical margins	0	–	
Mean follow-up (months)	26.1 ± 16.2	33.7 ± 22.9	0.25
Tumor persistence	0	1(6.25)	0.4
Local recurrence (%)	1(5)	3(18.75)	0.1
Metastasis	0	1(6.25)	0.4
Deaths	0	0	–

**Table 4:** Details on recurrences

	Recurrence type	n	Detection and treatment details
RFA	Local recurrence	3	One recurred on CT 8 months, re-ablated, now NED; the other two recurrence identified on CT at 12 months, then re-ablated, now NED.
	Metastatic recurrence	1	Two neoplasm were observed; then developed multiple metastasis at 13 months.
PN	Local recurrence	1	One detected at 6 months, being observed; then tried ablation in other hospital; still had segmental tumor residue, but was steady.

*RFA* radiofrequency ablation, *PN* partial nephrectomy, *NED* no evidence of disease, *CT* computer tomography

**Fig. 1:**
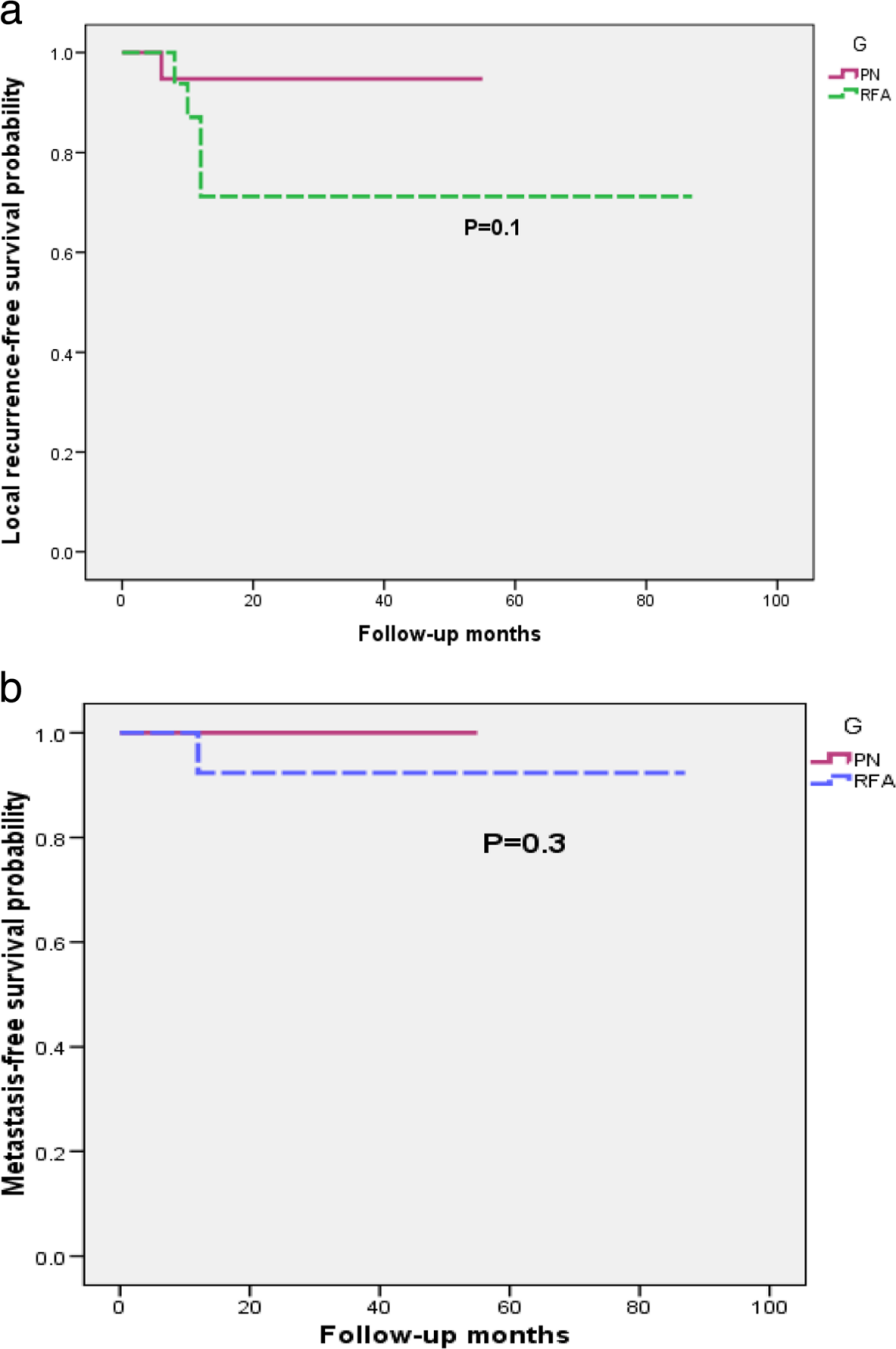
**a** and **b** local recurrence-free survival and metastasis-free survival for RFA versus PN

Figure 1a, b show the comparative outcomes for the local recurrence-free survival (RFS) and the metastasis-free survival (MFS) for PN group vs. RFA group. No significant difference between the two groups was indicated.

## Discussion

Tumor in a solitary kidney poses a distinct challenge for maintaining adequate renal function and providing oncologic control. PN is regarded as the gold standard treatment in patients with tumor in a solitary kidney [9], whereas RFA is an optional surgical procedure [10]. Both PN and RFA present certain advantages. Compared with PN, RFA showed easier recovery, less surgical trauma, less EBL, and a shorter length of hospitalization, thereby reducing recovery time and costs. These results were confirmed by Lotan Y and Cadeddu JA [11]. As shown in the study by Johnson DB et al. [12], RFA is a safe procedure with a low total complication rate (11.1%), which is similar to our findings (16.7%). Matsumoto ED et al. [13] indicated that tumors were successfully ablated in 100% of the cases when tumors < 3.7 cm and not centrally located. The total complication rate of PN seemed higher than that of RFA; however, no significant difference was found. This result is in agreement with the study by Wang Shangqian et al. [14], which demonstrated that total complications in PN treatment showed no difference from that in the RFA treatment. Similar to RFA, PN is a potentially safe method for a tumor in a solitary kidney.

Mean serum creatinine was significantly higher 24 h post-operatively than preoperatively in PN (*p* = 0.01), although this finding was not observed in RFA. Several studies indicated that RFA was better than PN because of the lower complication rate, particularly with respect to renal functional impairment. Johnson DB et al. [12] found that only 2% of patients with impaired renal function were examined again after RFA. Possible causes included the following: First, renal artery block was required in PN, which could impede renal function, whereas no hilar clamping was necessary in RFA. Research has shown that warm ischemia time > 30 min would cause irreversible damage to renal function [15]. Second, surgical trauma in PN was greater than that in RFA, which could aggravate damage to the kidney. Moreover, more renal parenchyma might be excised in PN. Three months post-operatively, no significant increase in the mean serum creatinine level was observed in PN, and neither PN nor RFA groups needed dialysis. PN and RFA exerted almost no effect on middle-term renal function. This finding suggested that both PN and RFA were safe procedures for a tumor in a solitary kidney. Jeffery W et al. [16] showed that adequate hydration, use of mannitol, minimization of the duration of renal ischemia, and involvement of a nephrologist in the perioperative care benefited the renal function.

No incidence of positive surgical margins was reported in PN in our database. Jeffery W et al. [16] showed that the positive surgical margin rate was 15%. The low positive surgical margin rate in the present study could improve by ultrasonic location when necessary during PN. Occurrence of positive margins predicts recurrence; research has shown that positive margin increases the risk of local recurrence as well as metastasis [17]. During follow-up, we observed 1 case (5%) of local recurrence in the PN group vs. 3 cases (18.75%) in the RFA group (*p* = 0.1). The local recurrent rate tended to be higher in the RFA group than in the PN group; however, no significant difference was indicated.

Our data and analysis showed that 2-year local RFS, MFS, and OS were statistically similar for RFA and PN. This finding was in agreement with the study by Ephrem O. Olweny et al. [18], who reported that 5-year OS and CSS, 5-year local RFS, MFS, and overall DFS were statistically similar for RFA and PN. Owing to its low local recurrent rate and long-term RFS, PN was an effective technique for tumor in a solitary kidney [1]. This finding was consistent with the data obtained in the current study, given that RFA obtained oncologic outcomes similar to those of PN. Therefore, both RFA and PN are effective approaches for the treatment of tumor in a solitary kidney.

The present study has certain limitations. (i) The study is a retrospective, nonrandomized design, which shows potential for selection bias and additional confounders. (ii) The small sample size weakened the statistical power of our analyses. (iii) Various methods for RFA (percutaneous, laparoscopic, open) and PN (open, laparoscopic, robot) could potentially influence our analyses. (iv) 4 patients being treated without a biopsy in the RFA group might make a difference to the recurrence rates. (v)The follow-up period was not sufficiently long to estimate long-term oncological and functional outcomes. Further evaluations require more cases and longer follow-ups.

## Conclusion

Our data showed that the oncological and functional outcomes of RFA and PN were similar, meaning that both RFA and PN are a safe and effective approach, which procedure is chosen depends on the common choice of patients and urologists. With the development of minimally invasive technology and the use of robots tumors that were difficult to excise once, such as in the renal pelvis or with huge size, can be successfully removed from a solitary kidney. For this reason an increasing number of specialists tend to select LPN or RPN (robotics-assisted partial nephrectomy). For patients who were old or whose physical health was poor, we prefer to recommend RFA.

## Abbreviations

CSS: Cancer-specific survival
DFS: Disease-free survival
EBL: Estimated blood loss
LPN: Laparoscopic partial nephrectomy
MFS: Metastasis-free survival
NSS: Nephron-sparing surgery
OPN: Open partial nephrectomy
OS: Overall survival
PN: Partial nephrectomy
RCC: Renal cell carcinoma
RFA: Radiofrequency ablation
RFS: Recurrence-free survival
RN: Radical nephrectomy
RPN: Robotics-assisted partial nephrectomy
